# Machine learning prediction of rapid HBsAg seroclearance at week 24 in inactive carriers treated with pegylated interferon

**DOI:** 10.1007/s12072-025-10936-x

**Published:** 2025-11-17

**Authors:** Jianxia Dong, Shan Ren, Pengxuan Wu, Haitian Yu, Xinyue Meng, Jing Zhao, Xiangyang Ye, Yan Huang, Zujiang Yu, Wenhua Zhang, Yilan Zeng, Xiaozhong Wang, Haibing Gao, Shuangsuo Dang, Jiabin Li, Sujun Zheng, Xinyue Chen

**Affiliations:** 1 The First Unit, Department of Hepatology, Beijing Youan Hospital, Capital Medical University, 8 Xitoutiao, Youan Men Wai, Fengtai District, Beijing, 100069 China; 2 The Affiliated Hospital of Putian University, Putian, China; 3 Xiangya Hospital of Central South University, Changsha, China; 4 The First Affiliated Hospital of Zhengzhou University, Zhengzhou, China; 5 Gansu Wuwei Tumor Hospital, Wuwei, China; 6 Public Health Clinical Center of Chengdu, Chengdu, China; 7 Xinjiang Uygur Autonomous Region Hospital of Traditional Chinese Medicine, Urumqi, China; 8 Mengchao Hepatobiliary Hospital of Fujian Medical University, Fuzhou, China; 9 The Second Affiliated Hospital of Xi’an Jiaotong University (Xibei Hospital), Xi’an, China; 10 The First Affiliated Hospital of Anhui Medical University, Hefei, China

**Keywords:** Inactive HBsAg carrier, Rapid HBsAg seroclearance, Machine learning, Prediction model, Light gradient boosting machine

## Abstract

**Background and aim:**

To identify predictive factors for rapid hepatitis B surface antigen (HBsAg) seroclearance at week 24 in inactive HBsAg carriers (IHC) receiving pegylated interferon alpha-2b (Peg-IFN) therapy, and to develop a machine learning-based model to optimize individualized treatment strategies.

**Methods:**

This retrospective analysis was based on a multicenter, prospective cohort study involving 2882 IHC patients treated with Peg-IFN and followed for at least 24 weeks. Predictive variables for week 24 HBsAg seroclearance were selected using both LASSO regression and the Boruta algorithm. Nine machine learning models were developed, including logistic regression (LR), decision tree (DT), and random forest (RF), with performance assessed via tenfold cross-validation. External validation was conducted in an independent cohort (*n* = 167) from three medical centers in Beijing. SHapley Additive Explanations (SHAP) were used to interpret model predictions and feature importance.

**Results:**

The overall HBsAg seroclearance rate at week 24 was 18.7% (541/2,882). Key predictive factors included baseline HBsAg level, ≥ 1 log IU/mL decline in HBsAg at week 12, the ratio of alanine aminotransferase (ALT) to HBsAg at week 12, the ratio of week 12 ALT to baseline HBsAg, week 12 hepatitis B virus (HBV) DNA level, and week 12 hepatitis B surface antibody (HBsAb) level. The Light Gradient Boosting Machine (Light GBM) model demonstrated the best performance, achieving an area under the receiver operating characteristic curve (AUC) of 0.902 (95% CI 0.881–0.923) and a sensitivity of 0.889 in the training cohort, and an AUC of 0.917 (95% CI 0.850–0.983) with a sensitivity of 0.879 in the external validation cohort. SHAP analysis revealed that the week 12 ALT/ HBsAg ratio was the most impactful feature.

**Conclusions:**

We developed a LightGBM-based machine learning model that accurately predicts rapid HBsAg seroclearance at week 24 among IHC patients receiving Peg-IFN therapy. This model offers a valuable tool for early identification of rapid responders, personalized treatment planning, and potential discontinuation strategies. The individualized stopping rules derived from model-predicted probabilities provide an evidence-based approach to precision therapy in IHC patients.

**Supplementary Information:**

The online version contains supplementary material available at 10.1007/s12072-025-10936-x.

## Introduction

Chronic HBV infection remains a major global public health concern. As of 2022, an estimated 254 million individuals live with chronic HBV infection worldwide. Viral hepatitis and its complications account for approximately 3500 deaths each day, with HBV being the leading cause [1]. Hepatitis B surface antigen (HBsAg) seroclearance is widely recognized as a hallmark of functional cure, which can significantly delay liver fibrosis progression, reduce the risk of hepatocellular carcinoma (HCC), and lower liver-related mortality [2]. Therefore, achieving HBsAg seroclearance has become a central goal in the treatment of chronic HBV infection.

IHCs represent a large yet historically underestimated subgroup of chronic HBV patients, particularly in regions of high endemicity such as Asia [3, 4]. Optimal management of this population holds substantial public health value for HBV elimination efforts. IHCs are typically characterized by persistent HBsAg positivity, undetectable serum HBV DNA levels, normal ALT level, and negative hepatitis B e antigen (HBeAg), reflecting suppressed viral replication. However, recent studies challenge the perception of IHCs as a “stable” state. Data from prospective liver biopsies have revealed that a considerable proportion of IHCs present with significant hepatic inflammation or fibrosis (grading ≥ 2 or staging ≥ 2) [5], suggesting potential disease progression and highlighting the need for therapeutic intervention. Long-term follow-up studies in Asian populations have further shown that IHCs have a 4.6-fold higher risk of HCC and a 2.1-fold higher risk of liver-related mortality compared to healthy individuals [6–8]. These findings underscore the clinical importance of considering treatment in IHCs to achieve HBsAg seroclearance.

Peg-IFN remains a key therapeutic option for inducing HBsAg seroclearance. Studies report that 48 weeks of Peg-IFN therapy in IHCs can achieve HBsAg seroclearance rates of 25% to 50% [9, 10], far exceeding the natural clearance rate of 1%–2% per year. However, host and viral factors—such as integrated HBV DNA and epigenetic silencing of covalently closed circular DNA (cccDNA)—may limit HBsAg seroclearance [11], resulting in 50–70% of patients failing to achieve this endpoint, while still being exposed to adverse effects and economic burden [12].

Among IHCs, achieving rapid HBsAg seroclearance within 24 weeks of Peg-IFN therapy holds particular clinical relevance. Early seroclearance often reflects stronger immune activation and viral suppression, and has been associated with lower treatment-related toxicity and improved cost-effectiveness [13]. Shorter treatment duration may reduce exposure to Peg-IFN-related side effects (e.g., fatigue, depression) and nucleos(t)ide analog (NA)-associated renal toxicity, thereby improving patient quality of life. According to the Global Burden of Disease (GBD) 2019 study, HBV-related disease accounted for over 23 million disability-adjusted life years (DALYs), with treatment-related adverse events contributing approximately 0.2–0.5 DALYs annually [14]. Shortening therapy by identifying early responders could therefore reduce DALYs and enhance both health and economic outcomes.

Our previous research and existing data suggest that more than half of eventual responders achieve HBsAg seroclearance within the first 24 weeks of therapy, highlighting the feasibility and value of early response prediction. Developing a prediction model for rapid HBsAg seroclearance at week 24 may enable timely identification of responders, allow individualized treatment adjustment, and provide a therapeutic window for precision management. However, most existing studies have focused on predictors of HBsAg seroclearance at 48 weeks, such as baseline HBsAg levels or early on-treatment HBsAg decline [13, 15], often requiring prolonged follow-up and failing to integrate dynamic multidimensional data [13, 15, 16]. To date, no studies have specifically addressed predictive modeling for HBsAg seroclearance within 24 weeks.

To address this gap, our study focuses on identifying predictors of rapid HBsAg seroclearance (within 24 weeks) and developing a dynamic, individualized prediction model using machine learning (ML). Compared with traditional statistical methods, ML can integrate high-dimensional clinical features and capture complex nonlinear relationships and variable interactions, thereby enhancing predictive performance [17]. Based on a multicenter, prospective cohort of IHC patients in China, we aimed to construct and validate an ML-based prediction model and provide a clinically applicable decision-support tool for optimizing Peg-IFN therapy in this population.

## Materials and methods

### Study population

This study was designed as a retrospective analysis based on data from multicenter prospective cohorts. The primary dataset was derived from the STARHB Project (Strategic Treatment and Research for Hepatitis B), a nationwide, real-world, prospective cohort study in China. The external validation dataset was obtained from an independent, multicenter, prospective, randomized controlled trial. Both cohorts followed a standardized research protocol, including baseline assessment, longitudinal follow-up, and efficacy evaluation (see Supplementary Figs. 1 and 2 for study design and workflow diagrams). Data from both high-quality prospective cohorts were integrated to construct the analytic dataset for model development and validation.

Inclusion criteria were based on the 2019 Chinese Guidelines for the Prevention and Treatment of Chronic Hepatitis B [18] and the EASL 2017 Clinical Practice Guidelines [4], and included: (1) age between 18 and 60 years; (2) persistent HBsAg positivity for > 6 months with quantitative HBsAg < 1000 IU/mL; (3) HBeAg-negative status; (4) sustained HBV DNA < 2000 IU/mL; and (5) persistently normal ALT (ALT < 40 U/L).

Exclusion criteria included: (1) incomplete 24-week follow-up (± 2 weeks allowed); (2) poor follow-up compliance (defined as fewer than two follow-up visits or loss to follow-up); (3) coinfection with hepatitis A virus (HAV), hepatitis C virus (HCV), hepatitis D virus (HDV), or human immunodeficiency virus (HIV); (4) use of immunomodulatory agents during treatment; (5) baseline hematological abnormalities (neutrophils < 1.5 × 10⁹/L, platelets < 90 × 10⁹/L); and (6) presence of autoimmune disease or uncontrolled thyroid dysfunction.

The external validation cohort strictly followed the same inclusion/exclusion criteria and follow-up protocols as the primary cohort and enrolled patients from three medical centers in Beijing. Baseline characteristics of this cohort are presented in Supplementary Table 2.

The study was approved by the local ethics committees at all participating centers (Primary cohort: No. 2022-067-K; External validation cohort: No. 2022-047-K), and conducted in accordance with the principles of the Declaration of Helsinki. The primary cohort was registered in the Chinese Clinical Trial Registry (ChiCTR2200061541), and the external validation cohort under ChiCTR2200059576. Written informed consent was obtained from all participants.

### Treatment protocol

All patients received Peg-IFN-based therapy. Those with baseline HBV DNA below the lower limit of detection were treated with Peg-IFN monotherapy, whereas patients with detectable HBV DNA received combination therapy with nucleos(t)ide analogs (NAs). A previous study [19] demonstrated no significant difference in 48-week HBsAg seroclearance rates between these regimens when stratified by baseline HBV DNA detectability. Peg-IFN was administered at a standard dose of 1.5 μg/kg once weekly by subcutaneous injection, with adjustments allowed based on individual HBsAg response and tolerability, and total treatment duration exceeding 24 weeks. First-line NAs recommended by current guidelines included entecavir (ETV), tenofovir disoproxil fumarate (TDF), tenofovir alafenamide (TAF), and tenofovir amibufenamide (TMF).

### Follow-up and data collection

All patients were evaluated at baseline (week 0) and every 12 weeks during treatment (± 7 days allowed).

Baseline data included demographic characteristics (age, sex, ethnicity, and HBV transmission route), laboratory results (HBV serologic markers, liver and renal function, complete blood count, HBV DNA, thyroid function and antibodies), and imaging assessments (abdominal ultrasound and liver stiffness measurement (LSM).

At each follow-up visit, laboratory tests, imaging, and safety evaluations were repeated.

The primary outcome was HBsAg seroclearance at week 24, defined as quantitative HBsAg < 0.05 IU/mL and HBV DNA < 20 IU/mL. Based on seroclearance status at week 24, patients were classified into a seroclearance group (CR) and a non-response group (NR).

### Data preprocessing

A three-level data quality control system was implemented: (1) variables with > 20% missing values were excluded; (2) continuous variables were subjected to outlier detection using the 1.5 × interquartile range (IQR) rule and Winsorized by truncating the top and bottom 1%; (3) missing values were imputed using random forest imputation (10 iterations, tree depth = 100).

Data normalization was performed by *Z *score transformation using the training set mean and standard deviation. Categorical variables were converted into dummy variables via one-hot encoding.

### Feature selection and collinearity control

A two-step feature selection strategy was employed. First, multicollinearity was addressed by removing variables with a variance inflation factor (VIF) > 5 or a Pearson correlation coefficient > 0.7. Second, key predictors were identified using both least absolute shrinkage and selection operator (LASSO) regression (tenfold cross-validation, 1-SE rule for λ selection) and the Boruta algorithm (500 iterations, *p* < 0.01). Features identified by both methods were retained. Feature stability was assessed using 1000 bootstrap resamples, and those selected in > 80% of iterations were included in final model development.

### Model development

We constructed predictive models using multiple machine learning algorithms: logistic regression (LR), decision tree (DT), random forest (RF), gradient boosting (GB), extreme gradient boosting (XGBoost), light gradient boosting machine (LightGBM), support vector machine (SVM), multilayer perceptron (MLP), and naïve Bayes (NB).

Model hyperparameters were tuned using Bayesian optimization, focusing on key parameters, such as number of trees (10–200), maximum depth (1–20), and minimum samples per split (2–20). The dataset was randomly split into a training set (70%) and a test set (30%) using stratified sampling to preserve distributional balance.

To address class imbalance, we applied threshold adjustment instead of using the default cutoff of 0.5. Specifically, the optimal probability threshold was determined within the training set by plotting receiver operating characteristic (ROC) curves and applying the Youden Index, in order to balance sensitivity and specificity. Model performance was evaluated using multiple metrics: AUC, accuracy, precision, sensitivity, specificity, and *F*1 score. SHAP algorithm was used to interpret feature importance and assess the contribution of individual variables. Optimized hyperparameters for each model are detailed in Supplementary Table 3.

### Statistical analysis

Continuous variables with normal distribution were expressed as mean ± standard deviation and compared using the Student’s *t *test. Non-normally distributed variables were reported as median (interquartile range) and compared using the Wilcoxon rank-sum test. Categorical variables were expressed as proportions and compared using the chi-square test or Fisher’s exact test, as appropriate. All analyses were performed using Python version 3.12.7 and R version 4.4.2. A two-sided *p *value < 0.05 was considered statistically significant.

## Results

### Baseline characteristics and treatment responses of the study cohort

#### Baseline characteristics

**Fig. 1:**
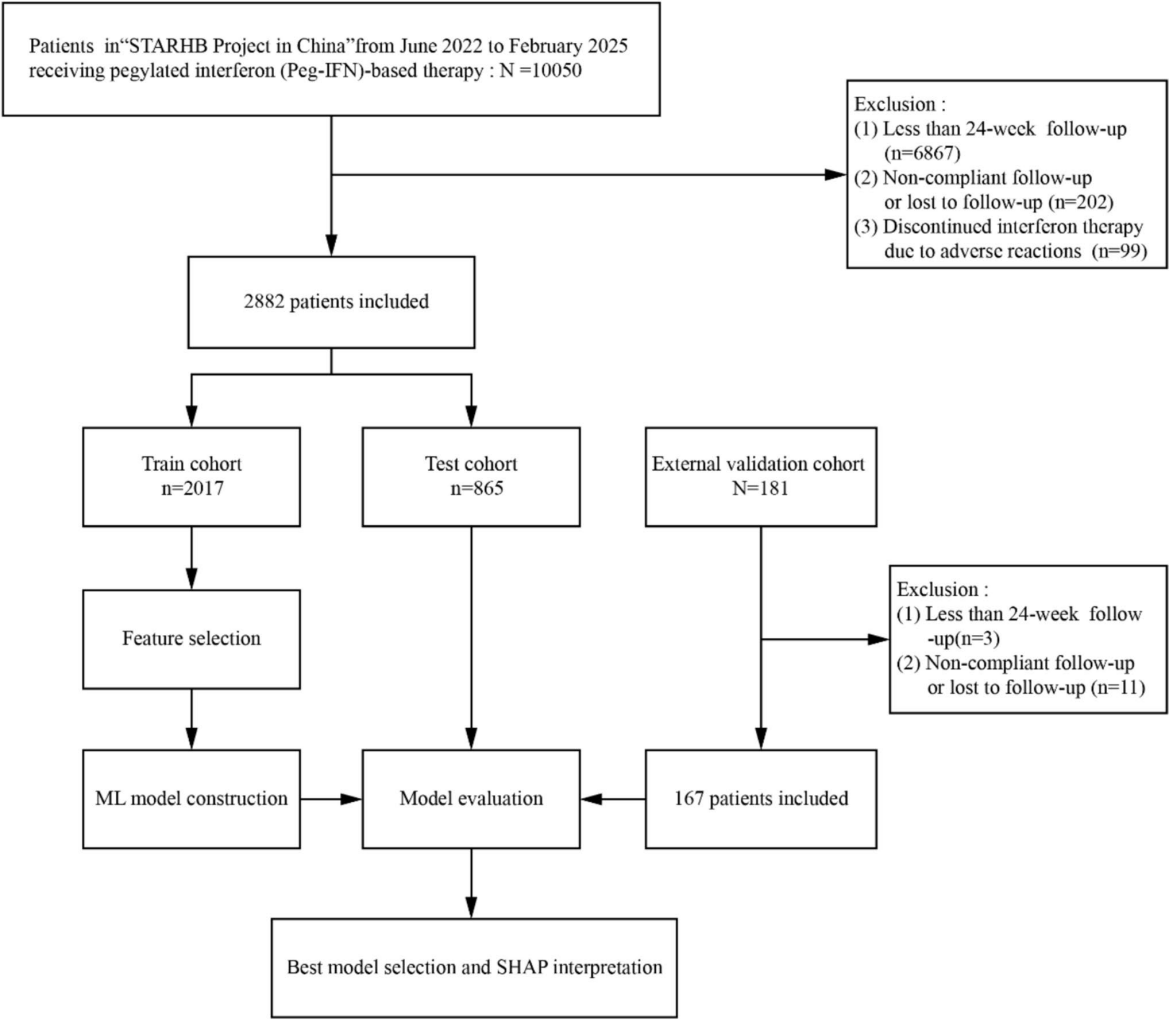
Flowchart of patient selection and model development

As shown in Fig. 1, a total of 2882 IHCs treated with either Peg-IFN monotherapy or in combination with NAs were included in this study. Patients were divided into an HBsAg seroclearance group (*n* = 541, 18.8%) and a non-clearance group (*n* = 2341, 81.2%) according to HBsAg seroclearance at week 24 of treatment.

Among the study population, 67.0% were male (1919/2882), the median age was 42 years (interquartile range [IQR]: 36–49 years), and 97.0% were of Han ethnicity (2783/2882). Non-maternal–infant transmission was predominant (91%). The median baseline hepatitis B virus (HBV) DNA level was 0 IU/mL (IQR: 0–136.75), indicating low viral load typical of IHC patients.

As shown in Table 1, patients in the HBsAg seroclearance group had significantly lower baseline HBsAg levels compared to the non-clearance group [median (IQR): 8.46 IU/mL (1.00–59.52) vs. 136 IU/mL (25.87–447.40), *p* < 0.001]. Additionally, the clearance group had slightly lower body mass index (BMI) [22.86 kg/m^2^ (21.82–24.22) vs. 23.18 kg/m^2^ (21.97–24.34), p = 0.02], and slightly higher white blood cell (WBC) counts [5.70 × 10⁹/L (4.73–6.72) vs. 5.49 × 10⁹/L (4.51–6.55), *p* = 0.01] and absolute neutrophil counts (ANCs) [3.21 × 10⁹/L (2.56–4.01) vs. 3.10 × 10⁹/L (2.42–3.91), *p* = 0.03]. Alpha-fetoprotein (AFP) levels were lower in the clearance group [2.66 ng/mL (2.03–3.24) vs. 2.75 ng/mL (2.04–3.56), *p* = 0.01], and the fibrosis-4 index (FIB-4) was also slightly lower [0.98 (0.72–1.32) vs. 0.99 (0.72–1.33), *p* = 0.02]; all differences were statistically significant.

**Table 1:** Baseline characteristics comparison between HBsAg loss and non-HBsAg loss groups at 24 weeks

Variables	Overall (*n* = 2882)	Non-HBsAg loss at 24-week (*n* = 2341)	HBsAg loss at 24-week (*n* = 541)	*p*
Baseline characteristics				
Male, *n* (%)	1919 (67)	1558 (67)	361 (67)	0.98
Age, years	42 (36, 49)	43 (36, 50)	40 (35, 47)	< 0.001
Nation, *n* (%)				0.99
Han	2783 (97)	2261 (97)	522 (96)	
Others	99 (3)	80 (3)	19 (4)	
BMI, kg/m^2^	23.12(21.97,24.33)	23.18(21.97, 24.34)	22.86 (21.82, 24.22)	0.02
Transmission, n (%)				0.51
Non-vertical	2613 (91)	2117 (90)	496 (92)	
Vertical	269 (9)	224 (10)	45 (8)	
Treatment, n (%)				0.08
PEG-IFNα-2b + NAs	1348 (47)	1114 (48)	234 (43)	
PEG IFNα-2b	1534 (53)	1227 (52)	307 (57)	
HBV DNA, IU/mL	0 (0, 136.75)	0 (0, 138)	0 (0, 132)	0.89
HBsAg, IU/mL	88.33(12.31,377.33)	136 (25.87,447.4)	8.46 (1,59.52)	< 0.001
HBsAb, IU/L	0 (0, 0.13)	0 (0, 0.13)	0 (0, 0)	0.91
HBeAg, COI	0.07 (0.01, 0.33)	0.07 (0.01, 0.33)	0.08 (0.01, 0.35)	0.59
ALT, U/L	21 (16, 27)	21.1 (16, 27.5)	20.8 (15.5, 26)	0.07
AST, U/L	21.2 (18, 25)	21.4 (18, 25)	21 (18, 24.1)	0.17
TBIL, μmol/L	13.5 (10.1, 17.77)	13.5 (10.16, 17.73)	13.3 (9.96, 17.8)	0.63
TP, g/L	74.2 (71.3, 77.2)	74.3 (71.4, 77.3)	74.2 (71.1, 77)	0.28
ALB, g/L	46.2 (44.12, 48.1)	46.2 (44.2, 48)	46.2 (44, 48.1)	0.96
BUN, mmol/L	4.99 (4.3, 5.63)	5 (4.29, 5.65)	4.95 (4.33, 5.57)	0.53
DBIL, μmol/L	3.5 (2.6, 4.98)	3.55 (2.6, 5)	3.5 (2.5, 4.9)	0.41
HB, g/L	150 (137, 160)	150 (137, 160)	149 (137, 160)	0.70
WBC, × 10^9^/L	5.53 (4.54, 6.6)	5.49 (4.51, 6.55)	5.7 (4.73, 6.72)	0.01
ANC, × 10^9^/L	3.13 (2.45, 3.94)	3.1 (2.42, 3.91)	3.21 (2.56, 4.01)	0.03
PLT, × 10^9^/L	205 (168, 242)	205 (168, 242)	207 (170, 242)	0.42
AFP, ng/ml	2.72 (2.04, 3.49)	2.75 (2.04, 3.56)	2.66 (2.03, 3.24)	0.01
LSM, kPa	6.65 (5.91, 8.81)	6.65 (5.93, 8.8)	6.69 (5.89, 8.9)	0.94
APRI	0.26 (0.2, 0.35)	0.26 (0.2, 0.35)	0.25 (0.2, 0.34)	0.16
FIB-4	0.98 (0.72, 1.32)	0.99 (0.72, 1.33)	0.94 (0.69, 1.23)	0.02
Characteristics at week 12				
HBsAg, IU/mL	31.54 (2, 202.49)	57.02(7.27, 280.54)	0.22 (0.01, 6.47)	< 0.001
HBsAg decline, log_10_/IU/mL	0.26 (-0.03, 1.04)	0.18 (-0.05, 0.7)	1.28 (0.39, 2.38)	< 0.001
HBsAb, IU/L	0.64 (0, 2.46)	0.54 (0, 1.95)	2.13 (0.03, 6.75)	< 0.001
HBV DNA, IU/mL	0 (0, 21.36)	0 (0, 24.5)	0 (0, 11.08)	< 0.001
ALT, U/L	58 (40, 84.4)	57.77 (40, 85.9)	59 (41, 77.68)	0.82
ALT rise	2.72 (1.8, 4.35)	2.72 (1.78, 4.38)	2.73 (1.88, 4.31)	0.39

*HBsAg* Hepatitis B Surface Antigen, *HBV DNA* Hepatitis B Virus Deoxyribonucleic Acid, *ALT* Alanine Aminotransferase, *AST* Aspartate Aminotransferase, *TBIL* Total Bilirubin, *TP* Total Protein, *ALB* Albumin, *Cr* Creatinine, *DBIL* Direct Bilirubin, *HB* Hemoglobin, *WBC* White Blood Cell Count, *ANC* Absolute Neutrophil Count, *PLT* Platelet Count, *AFP* Alpha-Fetoprotein, *HBeAg* Hepatitis B e Antigen, *COI* Cutoff Index, *HBsAb* Hepatitis B Surface Antibody, *PEG-IFNα-2b* Pegylated Interferon Alpha-2b, *NAs* Nucleoside/Nucleotide Analogs, *BMI* Body Mass Index, *ULN* Upper Limit of Normal, *ANC* Absolute Neutrophil Count, *AFP* Alpha-Fetoprotein, *LSM* Liver Stiffness Measurement

Continuous variables with normal distribution are compared using *t *tests, reported as mean ± SD. Non-normal continuous variables are compared using the Wilcoxon rank-sum test, reported as median (IQR). Categorical variables are expressed as percentages and compared using Fisher's exact tests

*p* < 0.05 was considered statistically significant

Other baseline characteristics—including sex, age, ethnicity, transmission route, treatment regimen (Peg-IFN ± NAs), HBV DNA, HBsAb, HBeAg, ALT, aspartate aminotransferase (AST), total bilirubin (TBIL), total protein (TP), albumin (ALB), blood urea nitrogen (BUN), creatinine (Cr), direct bilirubin (DBIL), hemoglobin (HB), platelet count (PLT), LSM, and AST to platelet ratio index (APRI)—showed no statistically significant differences between groups (all *p* > 0.05).

#### Characteristics of week 12 response

At week 12 of treatment, patients in the HBsAg seroclearance group demonstrated significantly stronger early virological and immunological responses. As shown in Table 1, the HBsAg level was significantly lower than that in the non-clearance group [0.22 IU/mL (0.01–6.47) vs. 57.02 IU/mL (7.27–280.54), p < 0.001], and the decline in HBsAg was more pronounced [1.28 log₁₀ IU/mL (0.39–2.38) vs. 0.18 log₁₀ IU/mL (− 0.05–0.70), *p* < 0.001]. HBV DNA levels were also lower [0 IU/mL (0–11.08) vs. 0 IU/mL (0–24.50), *p* < 0.001]. Furthermore, HBsAb levels at week 12 were significantly higher in the clearance group [2.13 IU/L (0.03–6.75) vs. 0.54 IU/L (0–1.95), *p* < 0.001], suggesting a more robust immune response. ALT levels [59 U/L (41–77.68) vs. 57.77 U/L (40–85.9), *p* = 0.82] and fold changes in ALT [2.73 (1.88–4.31) vs. 2.72 (1.78–4.38), *p* = 0.39] showed no significant differences between the groups.

#### External validation cohort characteristics

The external validation cohort included 167 patients, of whom 33 (19.8%) achieved HBsAg seroclearance at week 24. According to Supplementary Table 2, there were no significant differences in key baseline variables—such as sex (male: 58.7%), median age (42 years), HBV DNA [0 IU/mL (0–152)], HBsAg [110 IU/mL (15.2–350)], and ALT [21 U/L (16–27)]—when compared with the training set (*n* = 2017) and test set (*n* = 865) (all *p* > 0.05), indicating overall comparability across cohorts.

At week 12, the median HBsAg level in the external validation cohort was 26.9 IU/mL (IQR: 1.46–144), HBV DNA was 0 IU/mL (0–21), and ALT was 55 U/L (39–82); none of these showed significant differences compared with the training and test sets (all p > 0.05). The 24-week HBsAg seroclearance rates in the training, test, and external cohorts were 18.8%, 18.7%, and 19.8%, respectively (*p* = 0.95), supporting good external validity and generalizability of the model.

### Feature selection

To identify potential predictors of rapid hepatitis B surface antigen (HBsAg) seroclearance at 24 weeks, we conducted univariate logistic regression analysis in the training set to preliminarily screen candidate variables (*p* < 0.05). Subsequently, we applied two complementary feature selection methods: least absolute shrinkage and selection operator (LASSO) regression and Boruta algorithm, to reduce dimensionality and eliminate collinearity among variables. LASSO regression was performed using the “glmnet” package in R (version 4.2.2), with optimal regularization parameters determined by tenfold cross-validation. The Boruta algorithm, a wrapper built around a random forest (RF) classifier, was implemented using the “Boruta” package to iteratively assess variable importance and retain relevant features.

**Fig. 2:**
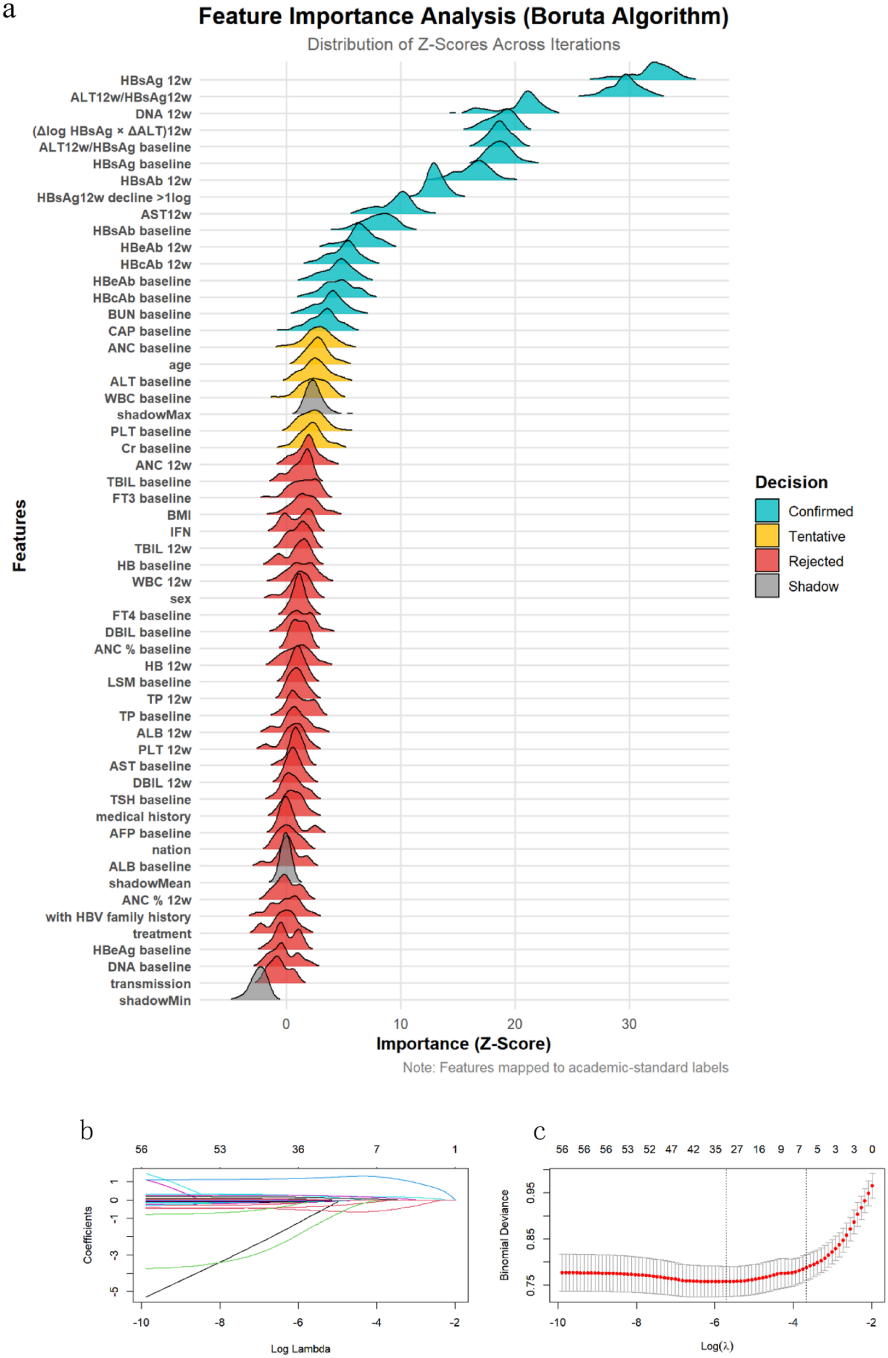
Feature selection by two algorithms. **a** Boruta algorithm feature selection. Confirmed (green): Key predictors; Rejected (red): Non-significant; Shadow (gray): Synthetic references; Tentative (orange): Uncertain features. **b** LASSO regression coefficient profiles of variables in the training dataset. **c** Selection of the optimal parameter (lambda) in the LASSO regression. *HBsAg 12w* HBsAg level at week 12, *ALT12w/HBsAg 12w* Ratio of ALT to HBsAg at week 12, *ALT12w/HBsAg baseline* Ratio of ALT at week 12 to baseline HBsAg, *HBsAg baseline* Baseline HBsAg level, *(Δlog HBsAg × ΔALT)12w* Product of log decline in HBsAg and increase in ALT at week 12, *HBsAg 12w decline > 1log* Whether HBsAg decline exceeds 1 log IU/mL at week 12, *DNA 12w* HBV DNA level at week 12, *HBsAb 12w* HBsAb level at week 12, *nation* Nationality of the patient, *ALT 12w up* Increase in ALT at week 12, *ALT baseline* Baseline ALT level, *ANC baseline* Baseline ANC, *TBIL baseline* Baseline *TBIL level, DNA baseline* Baseline HBV DNA level, *HB 12w* HB level at week 12, *ANC % baseline* Baseline ANC percentage, *HB baseline* Baseline HB level, *TP baseline* Baseline TP level, *ANC % 12w* ANC percentage at week 12, *WBC baseline* Baseline WBC, *AFP baseline* Baseline AFP level, *HBeAg baseline* Baseline HBeAg level, *HBsAb baseline* Baseline HBsAb level, *HBcAb baseline* HBcAb level at baseline, *HBeAb baseline* Baseline HBeAb level, *DBIL baseline* Baseline DBIL level, *TP 12w* TP level at week 12, *Cr baseline* Baseline Cr level, *PLT 12w* PLT at week 12, *AST baseline* Baseline AST level, *transmission* Transmission mode, *TBIL 12w* TBIL level at week 12, *sex* Sex of the patient, *WBC 12w* WBC at week 12, *DBIL 12w* DBIL level at week 12, *ANC 12w* ANC at week 12, *ALB baseline* Baseline ALB level, *ALB 12w* ALB level at week 12, *PLT baseline* Baseline PLT, *medical history* the time of HBV infection, *familyhistory* Family History of Hepatitis B Infection, *shadowMin* Minimum shadow feature value (used for algorithm comparison), *shadowMax* Maximum shadow feature value (used for algorithm comparison)

Variables identified by either LASSO or Boruta were further reviewed for clinical interpretability and redundancy. Highly correlated variables (Pearson’s *r* > 0.85) were considered for exclusion to avoid model overfitting. The final set of features selected by this two-step method was used to construct all subsequent machine learning (ML) models (see Fig. 2).

### Construction and evaluation of machine learning models

#### Model performance comparison

**Fig. 3:**
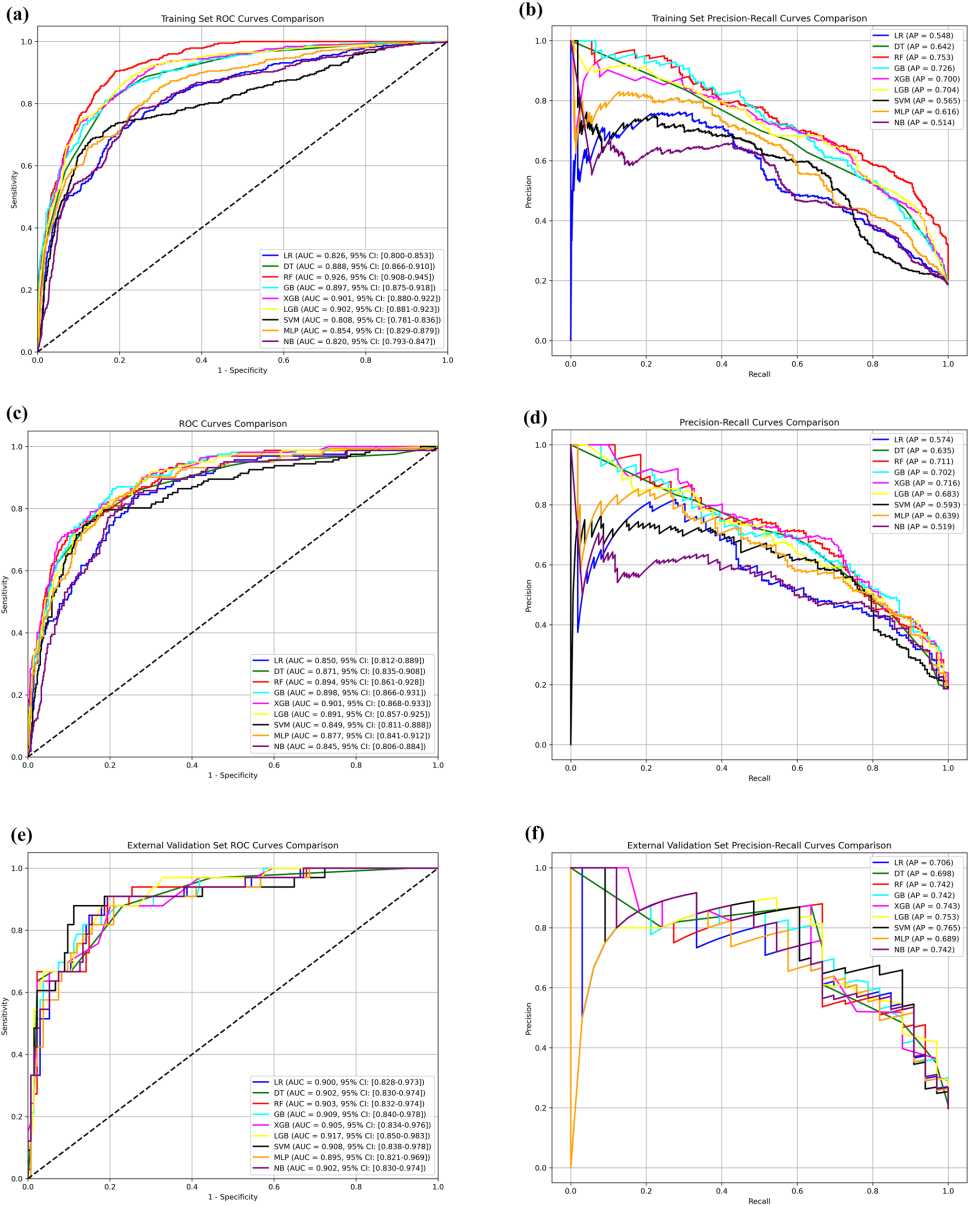
Model performance evaluation: a–b. Training Set: ROC Curves (Left) and Precision–Recall Curves (Right). c–d. Internal Testing Set: ROC Curves (Left) and Precision–Recall Curves (Right). e–f. External Validation Set: ROC Curves (Left) and Precision–Recall Curves (Right)

Based on the training set (*n* = 2017), internal testing set (*n* = 865), and external validation set (*n* = 167), we evaluated the performance of nine commonly used machine learning (ML) algorithms: logistic regression (LR), decision tree (DT), random forest (RF), gradient boosting (GB), extreme gradient boosting (XGBoost), light gradient boosting machine (LightGBM, LGB), support vector machine (SVM), multilayer perceptron (MLP), and naïve Bayes (NB). Model performance metrics are summarized in Tables 2, 3, and 4 and Fig. 3.

**Table 2:** Performance comparison of different machine learning models in the training set

Model	AUC	Accuracy	Precision	Sensitivity	Specificity	*F*1 Score
LR	0.826	0.779	0.446	0.720	0.793	0.551
DT	0.888	0.822	0.516	0.810	0.824	0.630
RF	0.926	0.827	0.524	0.905	0.810	0.663
GB	0.897	0.819	0.511	0.831	0.816	0.633
XGB	0.901	0.780	0.457	0.889	0.755	0.603
LGB	0.902	0.792	0.472	0.889	0.770	0.617
SVM	0.808	0.832	0.543	0.689	0.866	0.607
MLP	0.854	0.739	0.406	0.839	0.716	0.547
NB	0.820	0.758	0.422	0.784	0.752	0.548

*LR* Linear Regression, *DT* Decision Tree, *RF* Random Forest, *GB* Gradient Boosting, *XGB* eXtreme Gradient Boosting, *LGB* Light Gradient Boosting Machine, *SVM* Support Vector Machine, *MLP* Multilayer Perceptron, *NB* Naive Bayes

**Table 3:** Performance comparison of different machine learning models in the testing set

Model	AUC	Accuracy	Precision	Sensitivity	Specificity	*F*1 Score
LR	0.850	0.763	0.431	0.833	0.747	0.568
DT	0.871	0.827	0.525	0.772	0.839	0.625
RF	0.894	0.850	0.575	0.753	0.872	0.652
GB	0.898	0.807	0.491	0.864	0.794	0.626
XGB	0.901	0.837	0.545	0.790	0.848	0.645
LGB	0.891	0.806	0.489	0.827	0.801	0.615
SVM	0.849	0.843	0.560	0.747	0.865	0.640
MLP	0.877	0.756	0.428	0.901	0.723	0.581
NB	0.845	0.760	0.428	0.846	0.740	0.568

*LR* Linear Regression, *DT* Decision Tree, *RF* Random Forest, *GB* Gradient Boosting, *XGB* eXtreme Gradient Boosting, *LGB* Light Gradient Boosting Machine, *SVM* Support Vector Machine, *MLP* Multilayer Perceptron, *NB* Naive Bayes

**Table 4:** Performance comparison of different machine learning models in the external validation set

Model	AUC	Accuracy	Precision	Sensitivity	Specificity	*F*1 Score
LR	0.900	0.826	0.536	0.909	0.806	0.674
DT	0.902	0.790	0.483	0.879	0.769	0.624
RF	0.903	0.784	0.477	0.939	0.746	0.633
GB	0.909	0.856	0.600	0.818	0.866	0.692
XGB	0.905	0.814	0.518	0.879	0.799	0.652
LGB	0.917	0.832	0.547	0.879	0.821	0.674
SVM	0.908	0.886	0.659	0.879	0.888	0.753
MLP	0.895	0.814	0.517	0.909	0.791	0.659
NB	0.902	0.826	0.536	0.909	0.806	0.674

*LR* Linear Regression, *DT* Decision Tree, *RF* Random Forest, *GB* Gradient Boosting, *XGB* eXtreme Gradient Boosting, *LGB* Light Gradient Boosting Machine, *SVM* Support Vector Machine, *MLP* Multilayer Perceptron, *NB* Naive Bayes

In the training set (Table 2), most models achieved AUC values above 0.82. The RF model had the highest AUC (0.926) and sensitivity (0.905), but its precision was relatively low (0.524). The LGB model demonstrated a balanced performance with AUC of 0.902, sensitivity of 0.889, accuracy of 0.770, and *F*1 score of 0.617. Compared with GB (AUC = 0.897) and XGBoost (AUC = 0.901), the LGB model showed superior specificity (0.770) and precision (0.472), and it did not exhibit the low precision (0.516) observed in the DT model, indicating greater overall discriminative ability and stability.

In the testing set (Table 3), the LGB model maintained stable performance with an AUC of 0.891, accuracy of 0.806, sensitivity of 0.827, and *F*1 score of 0.615, outperforming other gradient boosting models. Although XGBoost achieved a slightly higher AUC (0.901), its precision (0.545) and *F*1 score (0.645) were comparable to LGB. The SVM model had high specificity (0.865), but lower sensitivity (0.747) and less consistent performance across datasets. The MLP model showed excellent sensitivity (0.901) but underperformed in precision and accuracy, resulting in a lower *F*1 score (0.581) compared to LGB.

In the external validation set (Table 4), the LGB model achieved the highest AUC (0.917), with accuracy of 0.832, *F*1 score of 0.674, sensitivity of 0.879, and specificity of 0.821—outperforming GB (AUC = 0.909) and XGBoost (AUC = 0.905). Although SVM had the highest accuracy (0.886) and *F*1 score (0.753) in the external set, its relatively low AUC in the training set (0.808) suggests potential overfitting to the validation cohort’s feature distribution. The RF model also showed high sensitivity (0.939) but low precision (0.477), with an *F*1 score of 0.633, falling short of the LGB model. ROC and precision–recall curves (Fig. 3) further confirm the consistent discriminative ability of the LGB model across datasets.

#### Final model selection

After comprehensive evaluation across all datasets, the LightGBM model was selected as the optimal predictive model for the following reasons:

Consistent performance: LGB maintained AUCs above 0.89 across the training, testing, and validation sets, with stable *F*1 scores ranging from 0.615 to 0.674 and no evidence of overfitting. Strong external validation: LGB achieved the highest AUC (0.917) and maintained balanced sensitivity (0.879) and specificity (0.821). Robust discriminative power: It offered balanced accuracy and *F*1 scores, performing well even in imbalanced data scenarios. Interpretability and clinical utility: LGB allows feature attribution analysis using SHAP, aiding clinical interpretation. Clinical benefit: Decision curve analysis (DCA) showed maximal net benefit within the threshold range of 0.2–0.8, with a low Brier score (0.091), indicating excellent calibration. Hence, LGB was ultimately adopted as the primary model in this study. Furthermore, its predictive efficacy was maintained in subgroup analyses stratified by treatment regimen (Supplementary Tables 4 and 5), demonstrating generalizability independent of concomitant NA therapy.

### Model interpretation

**Fig. 4:**
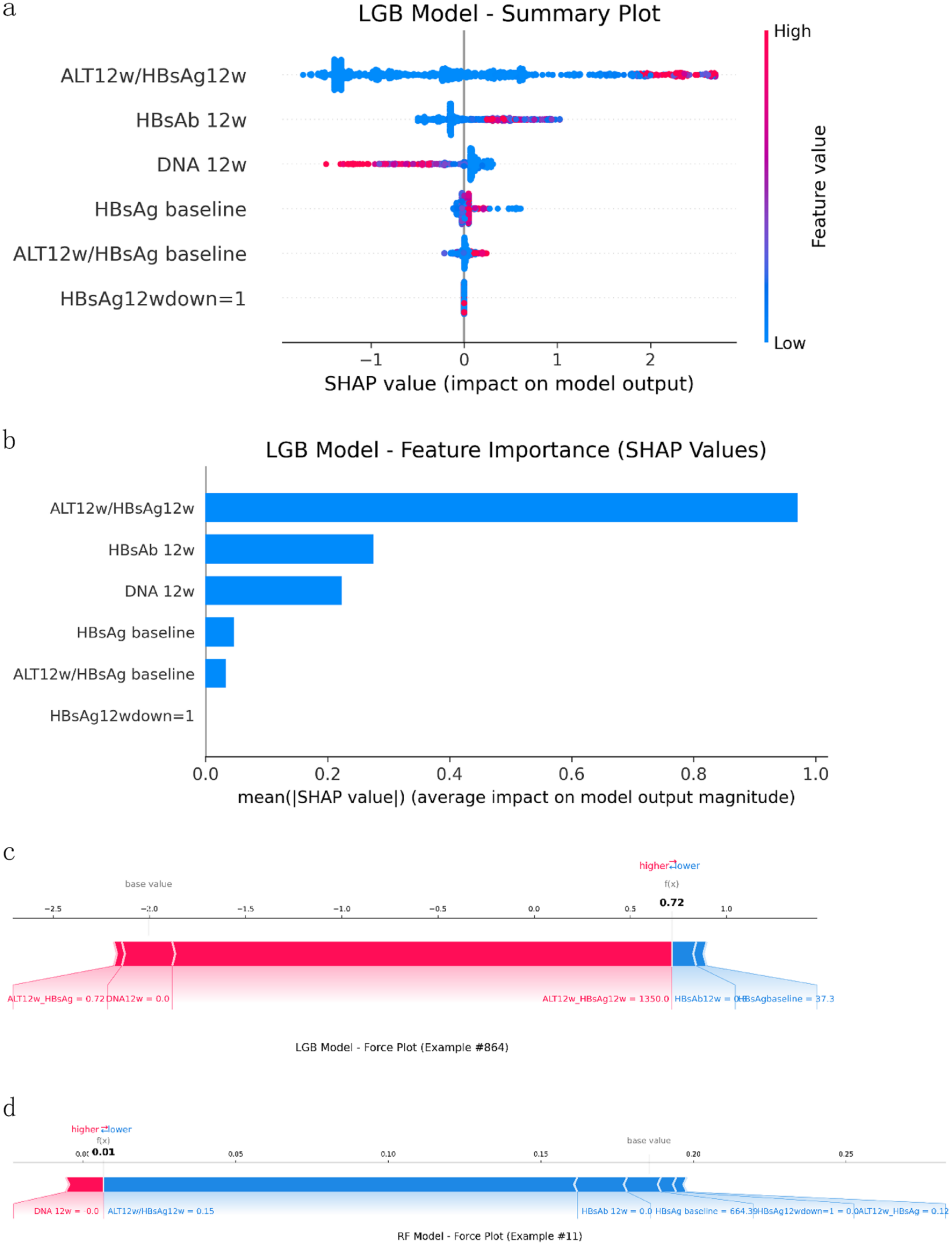
SHAP interpretation of the light GBM model: **a** SHAP summary plot, Positive SHAP values (right) boost the predicted probability of HBsAg clearance, while negative ones (left) lower it. Features are colored from low (blue) to high (red), illustrating how different value ranges affect predictions. **b** Feature importance ranking, reflecting their average influence on predicting HBsAg clearance. Higher values indicate greater feature importance. **c**, **d** SHAP force plots for individual predictions: individual contributions from patients with HBsAg loss (**c**) and (**d**) without HBsAg loss. The baseline (model's average output) serves as a reference. Positive contributions (red) raise the predicted HBsAg clearance probability, while negative ones (blue) reduce it

To further elucidate the prediction logic of the LGB model, we applied SHAP to perform both global and individual-level interpretation (Fig. 4).

#### Global feature importance

As shown in Fig. 4a, b, the most predictive feature in the LGB model was the ratio of alanine aminotransferase to hepatitis B surface antigen at week 12 (ALT12w/HBsAg12w), with a mean absolute SHAP value of 0.970—substantially higher than other variables. This was followed by hepatitis B surface antibody at week 12 (HBsAb12w, SHAP = 0.276), HBV DNA at week 12 (DNA12w, SHAP = 0.223), and baseline HBsAg level (HBsAg_baseline, SHAP = 0.047). The ratio of ALT12w to baseline HBsAg (ALT12w/HBsAg_baseline, SHAP = 0.034) also contributed moderately.

Interestingly, the variable “HBsAg decrease > 1 log IU/mL at week 12” (HBsAg12w_down = 1) had a SHAP value of 0, indicating limited independent predictive contribution, possibly due to multicollinearity with other dynamic markers.

SHAP directional analysis (Fig. 4a) revealed that a higher ALT12w/HBsAg12w ratio positively influenced the probability of HBsAg seroclearance. Similarly, elevated HBsAb12w and suppressed DNA12w levels contributed positively, reflecting enhanced immune response and viral suppression. In contrast, high baseline HBsAg levels had a negative impact on prediction outcomes (blue region shifted left). These trends align well with clinical observations in interferon-treated IHCs achieving rapid seroclearance.

#### Individualized Explanation

To illustrate the model’s individualized decision-making process, Fig. 4c, d presents SHAP local interpretation for two representative cases.

Patient 864 (Fig. 4c) achieved HBsAg seroclearance, with a predicted probability of 0.72. This prediction was driven by a high ALT12w/HBsAg12w ratio (1350), undetectable DNA12w (0 IU/mL), moderate ALT12w/HBsAg_baseline ratio (0.72), and > 1 log IU/mL decline in HBsAg at week 12. Despite negative HBsAb, the combined effect of these features significantly shifted the SHAP value to the right, illustrating the model’s integrated logic.

In contrast, Patient 11 (Fig. 4d) did not achieve seroclearance, with a predicted probability of only 0.01. This patient had a high baseline HBsAg (664.39 IU/mL), low ALT12w/HBsAg12w ratio (0.15), no significant HBsAg decline at week 12, negative HBsAb, and a low ALT12w/HBsAg_baseline ratio (0.12), all of which suppressed the prediction probability.

These findings highlight the LGB model’s ability to not only identify key predictors at the population level but also provide logical and interpretable insights at the individual level, especially emphasizing the predictive value of dynamic ALT–HBsAg ratios.

### Development of clinical decision tool

Based on the LGB model’s prediction probabilities, patients were stratified into low-, intermediate-, and high-risk groups (Supplementary Fig. 4). Except for the external validation cohort’s low- vs. intermediate-risk groups (*p* < 0.05), 24-week HBsAg seroclearance rates differed significantly among risk strata (*p* < 0.001). For clinical application, we recommend considering discontinuation of Peg-IFN treatment in low-risk patients (probability < 0.3) to reduce adverse effects and financial burden, while continuing therapy with dynamic evaluation in intermediate- and high-risk groups.

**Fig. 5:**
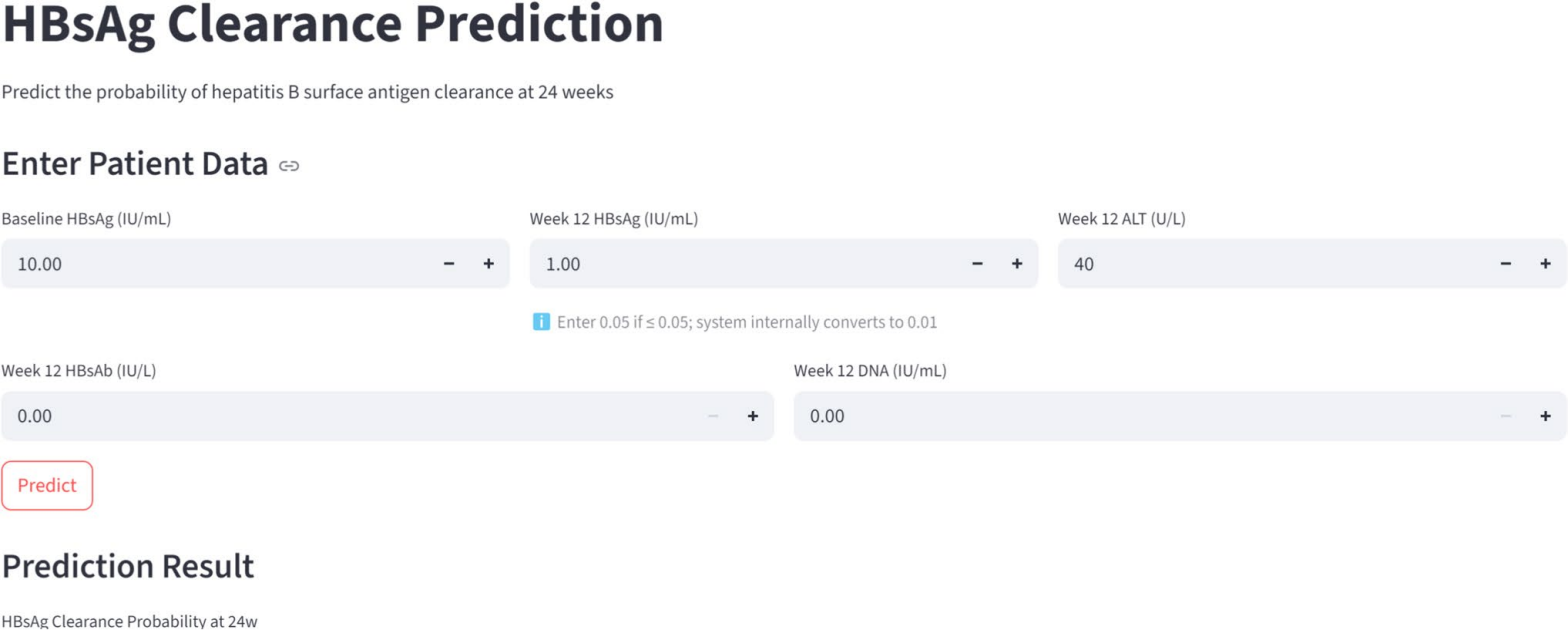
Web-based calculator interface for clinical application

Furthermore, we developed a web-based clinical prediction tool based on the LGB model (https://starplan-mkwvjd9gnio3zeon5qqrsn.streamlit.app/; interface shown in Fig. 5). By inputting individual values for baseline HBsAg, HBsAg12w, ALT12w, DNA12w, and HBsAb12w, the tool provides a real-time probability (0–100%) of achieving HBsAg seroclearance, offering practical support for personalized treatment strategies in IHC patients.

## Discussion

This study systematically analyzed predictors of rapid hepatitis B surface antigen (HBsAg) seroclearance within 24 weeks of Peg-IFN treatment in a large, multicenter, prospective cohort of IHCs (*n* = 2882). We developed and validated a Light Gradient Boosting Machine (LightGBM)-based machine learning model that demonstrated excellent predictive performance with AUC values of 0.902, 0.891, and 0.917 in the training, testing, and external validation cohorts, respectively. The model achieved balanced sensitivity (0.879) and specificity (0.821), outperforming traditional logistic regression approaches (AUC range 0.826–0.900) reported previously [13, 15, 19]. Key predictive factors identified included baseline HBsAg level, > 1 log IU/mL decline in HBsAg at week 12, the ratio of ALT to HBsAg at week 12, the ratio of ALT at week 12 to baseline HBsAg, HBV DNA level at week 12, and HBsAb level at week 12. Based on these factors, we also developed an online clinical prediction tool to provide personalized HBsAg seroclearance probabilities, aiding precision treatment decisions.

The six selected predictors construct a comprehensive framework for rapid HBsAg seroclearance prediction from three dimensions: viral load, treatment response, and immune activation. Low baseline HBsAg and significant HBsAg decline at week 12 have been validated in multiple studies [20]. A meta-analysis by Zhang et al. [13] confirmed low baseline HBsAg as a crucial predictor, reflecting the limiting effect of initial viral antigen burden on immune clearance. Chen et al. [15] further emphasized that early HBsAg decline indicates strong viral suppression and immune response, which are highly correlated with HBsAg seroclearance. Our findings align well with these observations, as shown by the significant contributions of these factors in the SHAP analysis.

The ratio of ALT to HBsAg at week 12 (ALT12w/HBsAg12w) emerged as the most influential dynamic predictor in our study, exhibiting the highest mean absolute SHAP value (0.970). This ratio reflects the balance between hepatocellular inflammatory activity (as indicated by ALT) and viral antigen burden (HBsAg) at week 12 of treatment, rather than highlighting ALT elevation alone. Elevated ALT levels are indicative of interferon-induced, immune-mediated hepatocyte injury, which mirrors the host’s clearance activity against infected cells [19, 21, 22]. Concurrently, lower HBsAg levels may mitigate immune suppression, promoting the recovery of T cell function [23, 24]. Within this framework, a higher ALT12w/HBsAg12w ratio is more likely to signify effective immune activation and antigen clearance—consistent with the immunological profile associated with HBsAg seroclearance [23]. Similarly, the ratio of ALT12w to baseline HBsAg (ALT12w/HBsAg_baseline) captures the interplay between early immune response and initial viral load. An elevated ratio implies more robust immune activation in the context of lower baseline HBsAg, which in turn favors HBsAg seroclearance [25, 26].

HBV DNA levels at week 12 represent viral replication suppression during mid-treatment. Low DNA levels typically indicate effective antiviral activity from NAs or interferon therapy, which may reduce immune escape pressure associated with viral replication and thus promote HBsAg seroclearance [4, 18, 27]. Previous studies have shown that under IFNα therapy and low viral load conditions, HBV may suppress transcription via mutations or epigenetic regulation of SP1/SP2 promoters, facilitating viral persistence [28]. Conversely, lower HBV DNA levels are more likely to disrupt this persistence mechanism by reducing basal core promoter (BCP) transcriptional activity, limiting replenishment of covalently closed circular DNA (cccDNA), thereby increasing the probability of HBsAg seroclearance. Our SHAP analysis corroborates this, showing a positive correlation between low HBV DNA at week 12 (SHAP = 0.223) and HBsAg seroclearance.

On the immune front, HBsAb reflects humoral immune response. Elevated HBsAb levels at week 12 (defined as absolute > 10 IU/L) indicate effective activation of humoral immunity, enhancing neutralization and clearance of circulating HBsAg [29, 30]. HBsAb not only binds and clears free hepatitis B viral particles, preventing infection of new hepatocytes, but may also promote antigen presentation via Fc receptor-mediated effector functions, activating T cell responses and potentially participating in infected hepatocyte clearance directly or indirectly [31]. Immune complexes formed by HBsAb and HBsAg can be phagocytosed by macrophages, further strengthening immune control. Studies have also shown that patients with low baseline HBsAg (≤ 1500 IU/mL) and HBsAb-specific B cells have higher rates of HBsAg seroclearance at end of treatment (EOT) [30–33]. These findings are consistent with our study’s observation that elevated HBsAb at week 12 (SHAP = 0.276) is significantly associated with HBsAg seroclearance. The median baseline HBsAb in our cohort was 0 IU/L, suggesting low initial humoral immune activity in IHC patients, and the rise in HBsAb post-treatment likely reflects Peg-IFN-induced immune activation, a key marker for rapid HBsAg seroclearance. Taken together, these virological and immunological markers form the biological basis of rapid HBsAg seroclearance and provide important early indicators for treatment response prediction.

Beyond its predictive role for Week 24 seroclearance, the clinical relevance of this early endpoint warrants attention. In our supplementary follow-up, rapid responders achieving HBsAg seroclearance by Week 24 demonstrated excellent durability: 91% (174/192) maintained clearance at the end of consolidation therapy (Week 48), and all evaluable patients sustained functional cure 24 weeks post-treatment (Week 72). At the longest follow-up (Week 96), 90% (9/10) remained in clinical cure, with only one case exhibiting minimal HBsAg rebound (0.07 IU/mL) while retaining HBeAg negativity and undetectable HBV DNA. These data indicate that early HBsAg seroclearance is a strong predictor of sustained functional cure, likely enhanced by full-course Peg-IFN consolidation (≥ 48 weeks) ensuring profound viral suppression and robust immunomodulatory benefits.

With respect to long-term HCC risk, no incident cases were observed during follow-up (up to 96 weeks) although this duration is insufficient for definitive assessment. Consistent with previous meta-analyses and large cohort studies, HBsAg seroclearance—whether achieved early or late—represents the most powerful predictor of markedly reduced HCC incidence and liver-related mortality, primarily through resolution of chronic hepatic inflammation [34–36]. Nonetheless, the risk is not fully abolished, particularly in patients with advanced fibrosis or cirrhosis, underscoring the necessity for continued surveillance even after functional cure.

Machine learning algorithms offer advantages over traditional statistical methods in handling large-scale, multivariate data [17, 37]. We used LASSO regression and Boruta feature selection to screen 54 candidate variables, removing redundancy and capturing nonlinear relationships and interactions. The LightGBM model exhibited robust generalizability with AUCs ≥ 0.89 across datasets. SHAP interpretation quantified feature contributions and clarified the direction and magnitude of key variables such as ALT12w/HBsAg12w, rendering the model’s predictive logic transparent and accessible for clinical adoption. The online prediction tool lowers application barriers, allowing physicians to obtain individualized HBsAg seroclearance probabilities in real time, and supports consideration of Peg-IFN cessation in low-risk patients (predicted probability < 0.3) to minimize adverse effects and reduce economic burden, optimizing therapeutic decisions.

This study has several limitations. First, despite the prospective multicenter cohort design, the retrospective analysis may introduce selection bias. No significant difference in HBsAg seroclearance rates was found between combination therapy and monotherapy groups (*p* = 0.08), possibly due to the low HBV DNA levels (median 0 IU/mL). Subgroup analysis showed that among patients with HBV DNA < 20 IU/mL, monotherapy with Peg-IFN resulted in significantly higher HBsAg seroclearance than combination therapy (21.0% vs. 15.8%, *p* = 0.005); however, no difference was observed in the HBV DNA ≥ 20 IU/mL subgroup (18.5% vs. 20.1%, p = 0.509). Second, the low median FIB-4 index suggests that patients achieving HBsAg seroclearance at 24 weeks had a lower risk of fibrosis or stronger immune clearance capacity, consistent with the generally low fibrosis characteristic of IHCs (median liver stiffness measurement ~ 6.65 kPa). However, the small median difference (0.01) between groups likely limits clinical significance. Furthermore, generalizability may be constrained as the cohort primarily involved Chinese Han individuals, with external validation limited to three Beijing centers. Another limitation is the lack of HBV genotyping, particularly relevant given prior associations between genotype B and higher seroclearance rates compared to genotype C [31]. Furthermore, omission of key virological and immunological biomarkers (HBcrAg, HBV RNA, host immune profiling) restricts mechanistic insight into immune heterogeneity and treatment response. Finally, while Week24 HBsAg clearance served as the primary endpoint, the long-term predictive accuracy for sustained functional cure beyond Week 48 requires further study.

To address these limitations, future studies should build upon our early predictive model by incorporating virological and host factors, such as HBV genotype, HBcrAg, and immune genetic profiles. This will refine individualized prediction of functional cure in IHCs—a population with distinct immunological characteristics and favorable responses to interferon-based therapy. Prospective trials are warranted to validate model-guided strategies for optimizing interferon use, such as early discontinuation in low-probability responders, thereby minimizing unnecessary exposure and enhancing cost-effectiveness. Further multi-center, diverse-population studies will strengthen the generalizability of the model. Ultimately, this approach supports tailored treatment strategies in IHCs, promoting precision medicine and accelerating progress toward WHO's 2030 hepatitis elimination goals.

## Supplementary Information

Below is the link to the electronic supplementary material.

Supplementary file1 (DOCX 744 KB)

## Data Availability

The data that support the findings of this study are available from the corresponding author, Xinyue Chen, upon reasonable request. The data are not publicly available due to privacy or ethical restrictions.
