# Supplementary material for: Machine learning prediction of rapid HBsAg seroclearance at week 24 in inactive carriers treated with pegylated interferon
Source: Hepatol Int. 2025 Nov 17;19(6):1331–46. doi: 10.1007/s12072-025-10936-x (PMC12715036; doi:10.1007/s12072-025-10936-x)
Supplement: Supplementary file 1 — Supplementary file1 (DOCX 744 KB) [file 12072_2025_10936_MOESM1_ESM.docx]

Machine Learning Prediction of Rapid HBsAg Seroclearance at Week 24 in Inactive Carriers Treated with Pegylated Interferon

Jianxia Dong^1#^, Shan Ren^,1#^, Pengxuan Wu^1^, Haitian Yu^1^, Xinyue Meng^1^ Jing Zhao^1^, Xiangyang Ye^2^, Yan Huang^3^, Zhujiang Yu^4^, Wenhua Zhang^5^, Yilan Zeng^6^, Xiaozhong Wang^7^, Haibing Gao^8^, Shuangsuo Dang^9^, Jiabin Li^10^, Sujun Zheng^1*^, Xinyue Chen^1*^

# These authors contributed equally to this work.

* Corresponding authors

**Journal Name:** Hepatology International

**Affiliation:**
1 The First Unit, Department of Hepatology, Beijing Youan Hospital, Capital Medical University

2. Affiliated Hospital of Putian University

3. Xiangya Hospital of Central South University

4. The First Affiliated Hospital of Zhengzhou University

5. Gansu Wuwei Tumor Hospital

6. Public Health Clinical Center of Chengdu

7. Xinjiang Uygur Autonomous Region Hospital of Traditional Chinese Medicine

8. Mengchao Hepatobiliary Hospital of Fujian Medical University

9. The Second Affiliated Hospital of Xi'an Jiaotong University (Xibei Hospital)

10. The First Affiliated Hospital of Anhui Medical University

**Corresponding Author:** Xinyue Chen

**Contact Information for Corresponding Author:**

Email: chenxydoc@ccmu.edu.cn

Tel: +8613911212398

Address:The First Unit, Department of Hepatology, Beijing Youan Hospital, Capital Medical University,8 Xitoutiao, Youan men wai, Fengtai District, Beijing 100069, China

Supplementary table 1 Baseline characteristics comparison between training and testing sets

| Variables | Overall  (n = 2882) | Train set  (n = 2017) | Test set  (n = 865) | p |
| --- | --- | --- | --- | --- |
| Baseline characteristics |  |  |  |  |
| Age, years | 42 (36, 49) | 42 (36, 49) | 42 (35.93, 49) | 0.67 |
| Male,n (%) | 1919 (67) | 1341 (66) | 578 (67) | 0.90 |
| BMI, kg/m^2^ | 23.12 (21.97,24.33) | 23.15(21.97,24.34) | 23.04 (21.94, 24.3) | 0.61 |
| Nation, HAN,n (%) | 2783 (97) | 1946 (96) | 837 (97) | 0.79 |
| HBV DNA, IU/mL | 0 (0,136.75) | 0 (0, 142) | 0 (0, 121.9) | 0.67 |
| HBsAg, IU/mL | 88.33(12.31,377.33) | 89.4(12.28,388.06) | 87.2(12.9, 350) | 0.55 |
| HBsAb, IU/L | 0 (0, 0.13) | 0 (0, 0.07) | 0 (0, 0.28) | 0.48 |
| HBeAg, COI | 0.07 (0.01, 0.33) | 0.08 (0.01, 0.34) | 0.07 (0.01, 0.33) | 0.37 |
| ALT, U/L | 21 (16, 27) | 21.3 (16, 27.3) | 21 (16, 27) | 0.55 |
| AST, U/L | 21.2 (18, 25) | 21.1 (18, 25) | 21.5 (18, 25) | 0.91 |
| TBIL, μmol/L | 13.5 (10.1, 17.77) | 13.5 (10.2, 17.73) | 13.5 (10, 17.8) | 0.74 |
| TP, g/L | 74.2 (71.3, 77.2) | 74.2 (71.3, 77.1) | 74.3 (71.4, 77.2) | 0.47 |
| ALB, g/L | 46.2 (44.12, 48.1) | 46.2 (44.1, 48) | 46.2 (44.2, 48.2) | 0.39 |
| BUN, mmol/L | 4.99 (4.3, 5.63) | 5 (4.29, 5.62) | 4.97 (4.3, 5.67) | 0.97 |
| Cr, μmol/L | 73.48 (60.3, 80.5) | 73.98 (60.6, 80.49) | 72.96 (60, 80.55) | 0.41 |
| DBIL, μmol/L | 3.5 (2.6, 4.98) | 3.5 (2.6, 5) | 3.57 (2.6, 4.9) | 0.71 |
| WBC, ×10^9^/L | 5.53 (4.54, 6.6) | 5.5 (4.53, 6.61) | 5.61 (4.59, 6.54) | 0.74 |
| ANC, ×10^9^/L | 3.13 (2.45, 3.94) | 3.13 (2.45, 3.96) | 3.11 (2.46, 3.89) | 0.55 |
| HB, g/L | 150 (137, 160) | 149 (137, 160) | 150 (137, 160) | 0.29 |
| PLT, ×10^9^/L | 205 (168, 242) | 204 (167, 241.13) | 208 (170, 243) | 0.42 |
| AFP, ng/ml | 2.72 (2.04, 3.49) | 2.71 (2.03, 3.45) | 2.75 (2.07, 3.58) | 0.38 |
| Characteristics at week 12 |  |  |  |  |
| HBsAg, IU/mL | 31.54 (2, 202.49) | 33 (1.92, 197.72) | 28.79 (2.42, 203.8) | 0.94 |
| HBsAg decline,  log_10_/IU/mL | 0.26 (-0.03;1.04) | 0.26 (-0.03;1.04) | 0.25 (-0.03;1.03) | 0.81 |
| HBsAg decline  >log_10_/IU/mL, n (%) | 746 (26) | 522 (26) | 224 (26) | 0.99 |
| HBV DNA, IU/mL | 0 (0, 21.36) | 0 (0, 21.85) | 0 (0, 20) | 0.73 |
| ALT, U/L | 58.0 (40.0;84.4) | 57.9 (40.0;84.0) | 58.3 (41.0;85.0) | 0.69 |
| ALT rise | 2.72 (1.80;4.35) | 2.71 (1.79;4.33) | 2.76 (1.85;4.38) | 0.61 |

Supplementary table 2. Baseline characteristics comparison among training, testing, and external validation sets

| Variable | Train set  (n = 2017) | Test set  (n = 865) | External set  (n = 167) | P |
| --- | --- | --- | --- | --- |
| **Baseline characteristics** |  |  |  |  |
| Male, n(%) | 1341 (66.5) | 578 (66.8) | 98 (58.7) | 0.11 |
| age | 42 (36,49.) | 42 (35.;49) | 42(35,48) | 0.71 |
| HBV DNA, IU/mL | 0 (0, 142) | 0 (0, 122) | 0 (0.00, 152) | 0.92 |
| HBsAg, IU/mL | 89.4 (12.3,388) | 87.2 (12.9,350) | 110 (15.2,350) | 0.83 |
| ALT, U/L | 21.3 (16.0,27.3) | 21.0 (16.0,27.0) | 21.0 (16.0,27.0) | 0.83 |
| AST, U/L | 21.1 (18, 25) | 21.5 (18, 25) | 23.0 (19.0,25.4) | 0.16 |
| WBC, ×10^9^/L | 5.5 (4.53, 6.61) | 5.61 (4.59, 6.54) | 5.34 (4.60,6.28) | 0.67 |
| ANC, ×10^9^/L | 3.13 (2.45, 3.96) | 3.11 (2.46, 3.89) | 3.05 (2.50,3.82) | 0.83 |
| HB, g/L | 149 (137, 160) | 150 (137, 160) | 151 (137,159) | 0.57 |
| PLT, ×10^9^/L | 204 (167, 241.13) | 208 (170, 243) | 205 (169, 240) | 0.38 |
| **Characteristics at week 12** |  |  |  |  |
| HBsAg | 33.0 (1.92,198) | 28.8 (2.42,204) | 26.9 (1.46,144) | 0.84 |
| ALT, U/L | 57.9 (40,84) | 58.3 (41,85) | 55 (39, 82) | 0.78 |
| HBV DNA, IU/mL | 0.00 (0.00,21.9) | 0.00 (0.00,20.0) | 0.00 (0.00, 21.0) | 0.89 |
| HBsAg loss at week 24,  n (%) | 379 (18.8) | 162 (18.7) | 33 (19.8) | 0.95 |

Supplementary Table 3. The optimal hyper-parameters and metrics of machine learning models

| **Model** | **Hyperparameter** |
| --- | --- |
| **LR** | penalty=l2, C=1.0, max_iter=1000 |
| **DT** | random_state: 42, max_depth: 4, min_samples_leaf: 14 min_samples_split: 15 |
| **RF** | max_depth: 9, max_features: 0.994, min_samples_leaf: 18, min_samples_split: 9, n_estimators: 69, random_state: 42 |
| **GB** | learning_rate: 0.05, max_depth: 3, max_features: 0.430, min_samples_leaf: 9, min_samples_split: 16, n_estimators: 67, subsample: 0.76, random_state: 42 |
| **XGB** | learning_rate: 0.13, max_depth: 9, min_child_weight: 1, n_estimators: 46, subsample: 0.62, random_state: 42 |
| **LGB** | learning_rate: 0.19, max_depth: 13, min_child_samples: 47, n_estimators: 13, num_leaves: 9, reg_alpha: 0.48, reg_lambda: 0.86, subsample: 0.53, random_state: 42 |
| **SVM** | C: 0.98, kernel: rbf, |
| **MLP** | alpha: 0.094, learning_rate_init: 0.036, hidden_layer_sizes: (20, 11), max_iter: 1000, |
| **NB** | var_smoothing: 3.74546373446174e-06 |

Supplementary Table 4 Performance comparison of different machine learning models in in Peg-IFN and NAs subgroup

| **Model** | **AUC** | **Accuracy** | **Precision** | **Sensitivity** | **Specificity** | **F1 Score** |
| --- | --- | --- | --- | --- | --- | --- |
| **LR** | 0.852 | 0.803 | 0.459 | 0.769 | 0.810 | 0.575 |
| **DT** | 0.894 | 0.845 | 0.536 | 0.786 | 0.857 | 0.638 |
| **RF** | 0.926 | 0.838 | 0.521 | 0.868 | 0.832 | 0.651 |
| **GB** | 0.908 | 0.849 | 0.542 | 0.833 | 0.852 | 0.657 |
| **XGB** | 0.911 | 0.786 | 0.444 | 0.927 | 0.756 | 0.600 |
| **LGB** | 0.909 | 0.803 | 0.465 | 0.910 | 0.780 | 0.616 |
| **SVM** | 0.837 | 0.842 | 0.532 | 0.748 | 0.862 | 0.622 |
| **MLP** | 0.873 | 0.769 | 0.420 | 0.872 | 0.747 | 0.567 |
| **NB** | 0.835 | 0.793 | 0.444 | 0.769 | 0.798 | 0.563 |

Supplementary Table 5 Performance comparison of different machine learning models in Peg-IFN subgroup

| **Model** | **AUC** | **Accuracy** | **Precision** | **Sensitivity** | **Specificity** | **F1 Score** |
| --- | --- | --- | --- | --- | --- | --- |
| **LR** | 0.816 | 0.773 | 0.456 | 0.700 | 0.791 | 0.553 |
| **DT** | 0.874 | 0.802 | 0.503 | 0.811 | 0.800 | 0.621 |
| **RF** | 0.908 | 0.804 | 0.507 | 0.876 | 0.786 | 0.642 |
| **GB** | 0.887 | 0.808 | 0.513 | 0.814 | 0.807 | 0.630 |
| **XGB** | 0.892 | 0.852 | 0.605 | 0.749 | 0.878 | 0.670 |
| **LGB** | 0.889 | 0.799 | 0.499 | 0.850 | 0.786 | 0.629 |
| **SVM** | 0.807 | 0.809 | 0.517 | 0.713 | 0.833 | 0.599 |
| **MLP** | 0.849 | 0.756 | 0.440 | 0.795 | 0.747 | 0.566 |
| **NB** | 0.820 | 0.763 | 0.447 | 0.775 | 0.760 | 0.567 |


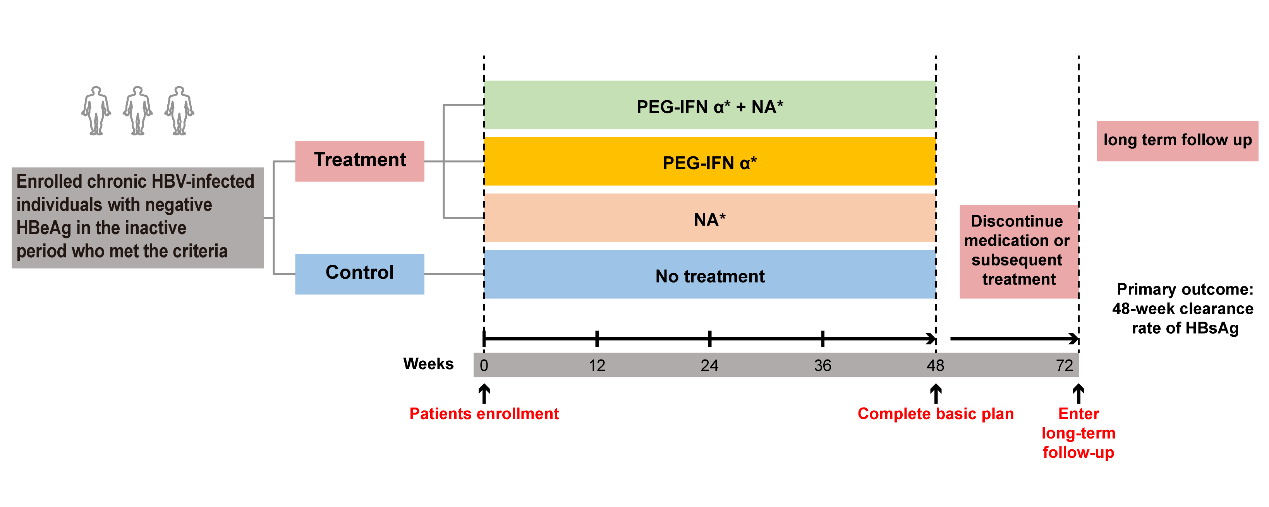


**Supplementary Fig1.** Research Protocol for the Chinese Hepatitis B Clinical Cure STAR Project


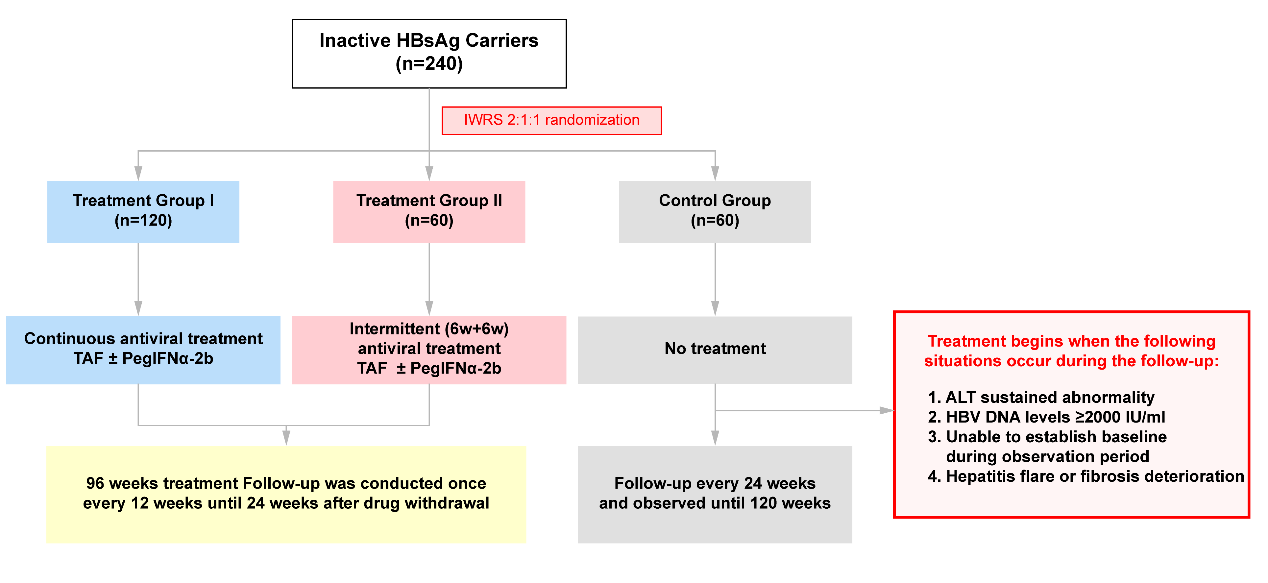


**Supplementary Fig2.** External Validation Cohort Study Protocol


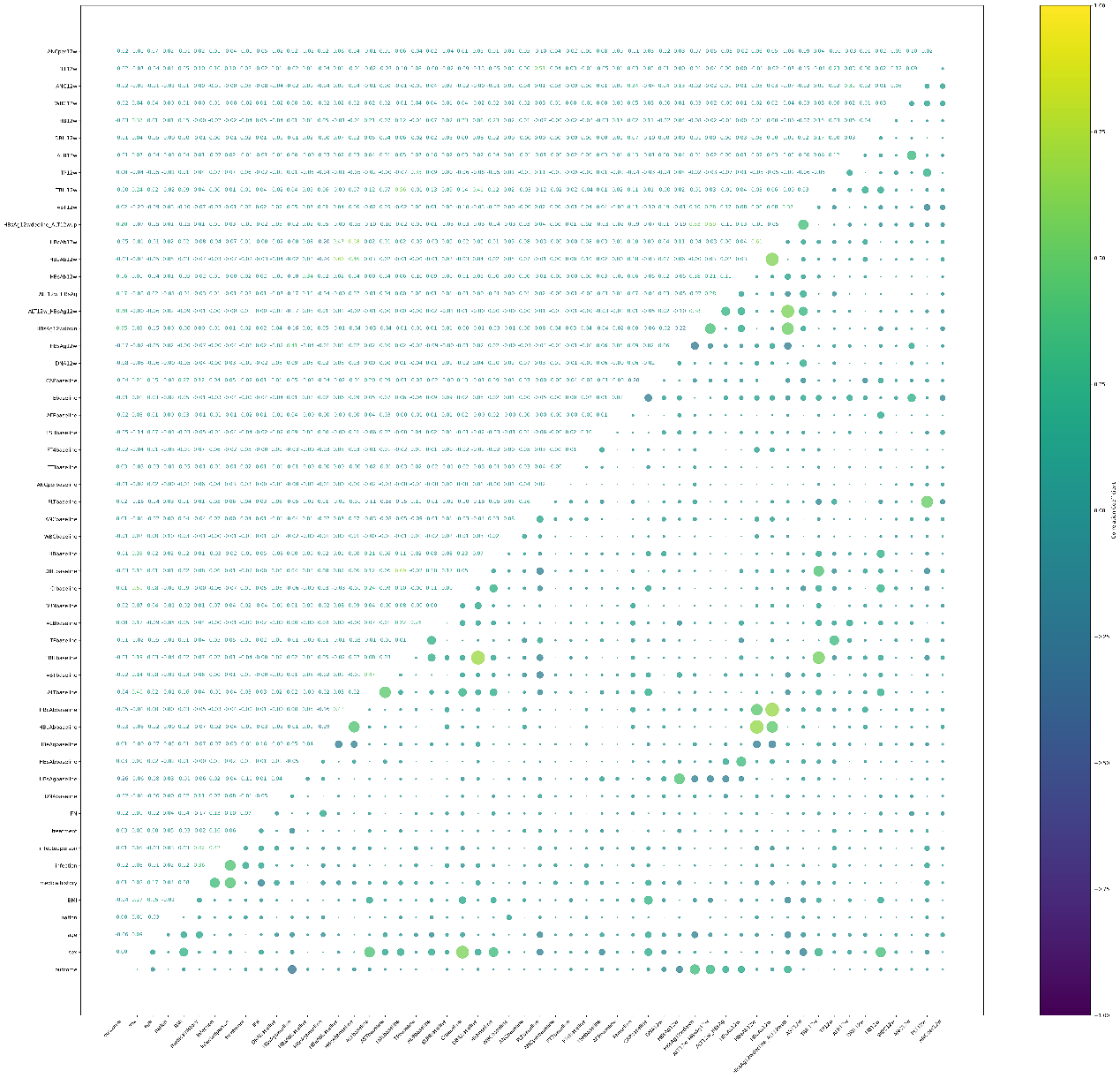


**Supplementary Fig3.** Feature correlation

a b


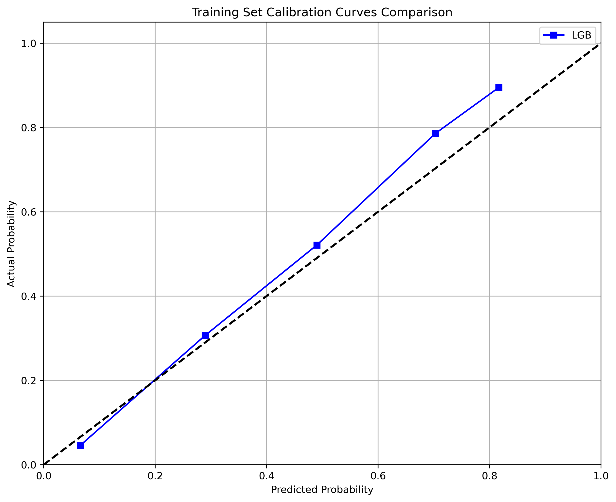

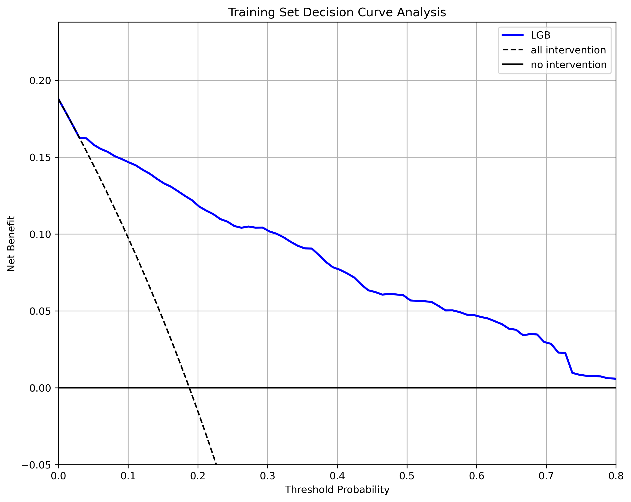


c d


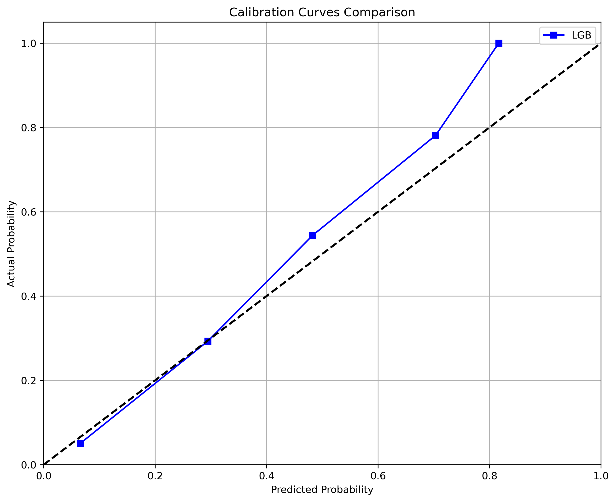

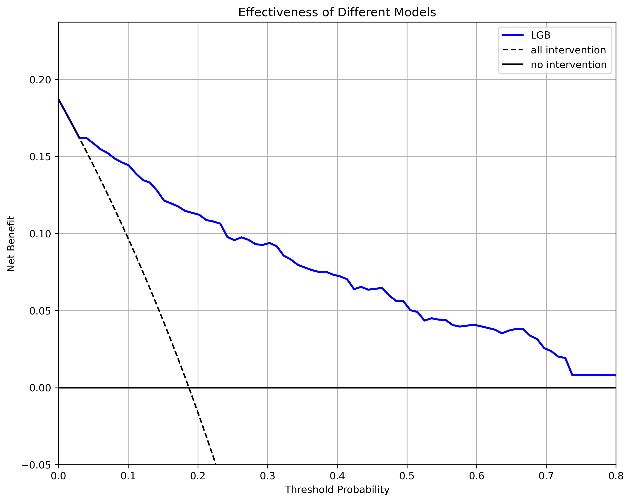


e f


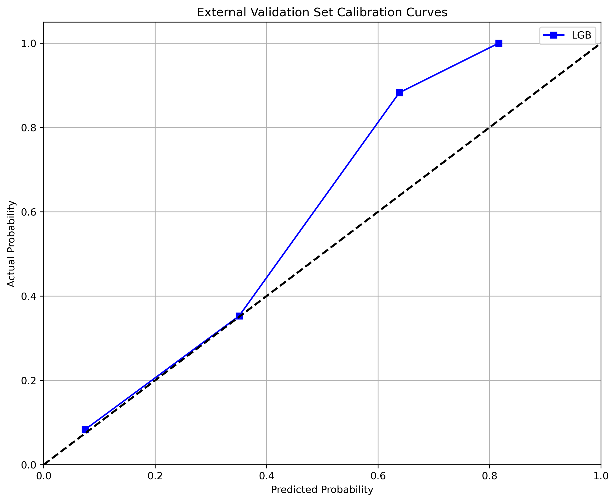

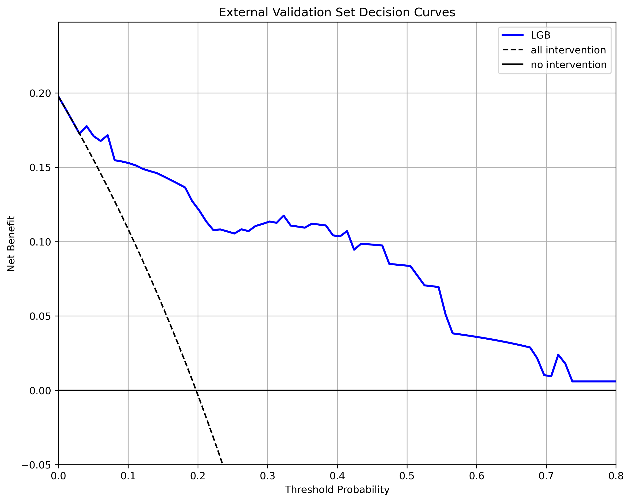


**Supplementary Fig4.** Calibration curves (left) and decision curves (right) for LGB model across different cohorts A-B. Calibration curves (left) and decision curves (right) . C-D. Internal Testing Set: Calibration curves (left) and decision curves (right) E-F. External Validation Set: Calibration curves (left) and decision curves (right).


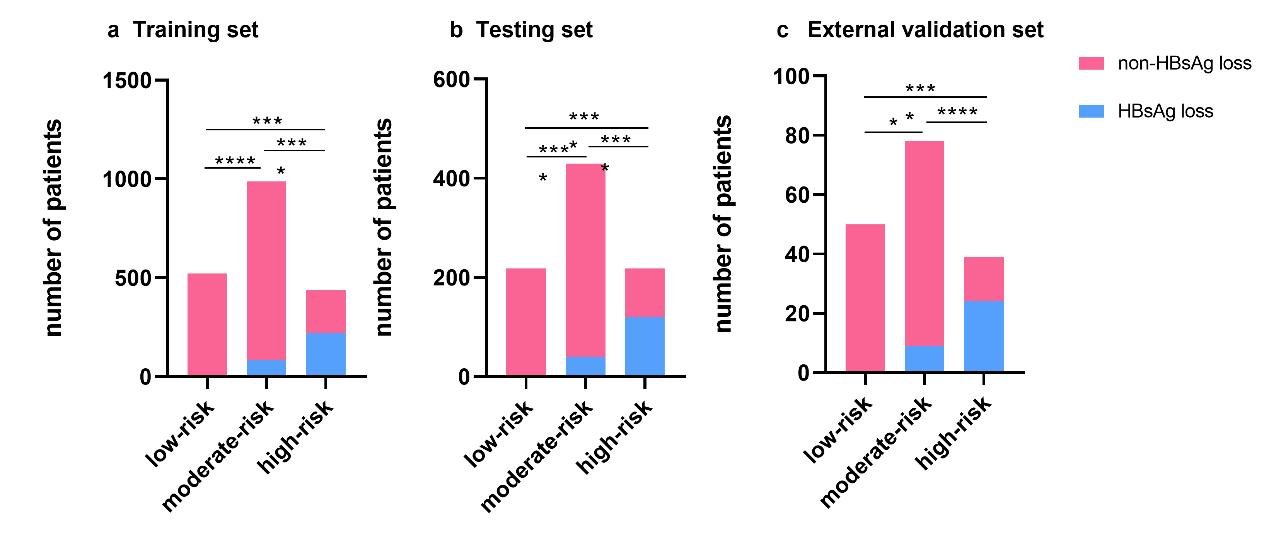


**Supplementary Figure5.** Distribution of HBsAg Clearance Status of 3 risk groups assessed by LGB model. (* p < 0.05, ** p < 0.01, *** p < 0.001, **** p < 0.0001)
